# Technology in education

**Published:** 2022-06-07

**Authors:** Michelle L Hennelly, Irene Ctori

**Affiliations:** 1 Divisional Lead: Optometry and Visual Sciences, City, University of London, UK.; 2 Associate Dean: Education Quality and Student Experience and Associate Professor: Division of Optometry and Visual Sciences, City, University of London, UK.

**During the pandemic, many eye care educators have turned to technology to deliver eye care education.**

The COVID-19 pandemic has triggered an unprecedented change in the delivery of eye care education. Before the pandemic, most education – ranging from continuing education and training (CET) to postgraduate training – was delivered as a combination of in-person lectures or tutorials and practical, clinical sessions. In March 2020, however, universities and colleges had to close their doors, and educators were forced to use online communication platforms to reach their students.

## What is online learning?

Online learning is a way of delivering educational material and classes over the internet, instead of in a face-to-face classroom setting, using an online, virtual environment to host and support education. Educational content is constructed using principles of teaching and learning (pedagogy) and supports student progression and success. Tools and technology are used to support delivery of the content, but its success is built on creating an inclusive, accessible, and interactive environment.

Online learning can improve access to education and flexibility for people who would otherwise have to travel long distances or struggle to balance studying with their work commitments. There are two main approaches to content delivery: **synchronous** and **asynchronous**.

**Synchronous** (‘live’) online learning experiences such as presenting live lectures or demonstrations, or leading small group discussions via video platforms such as Zoom or MS Teams, make it possible for people to take part in courses offered far away from where they live. Students share the same virtual space as their tutors and fellow students and can ask questions and interact in a range of different ways. However, a stable and reliable connection to the internet is essential and everyone has to be available to join sessions at the same time – which can be a challenge if students are based in different time zones, work different shift patterns, or have personal challenges (as was the case during the pandemic). Therefore, shorter presentations, recordings, and transcripts, which are typical of asynchronous learning, may better support engagement and learning amongst eye care professionals.

**Asynchronous** (‘at your own pace’) online learning takes place when students engage with study materials that are delivered online, such as lecture videos and notes, quizzes, question papers, and worked examples. Students work through content at their own pace. They can also can post questions and comments on discussion boards, allowing them to interact with educators, subject experts, and fellow students. This approach facilitates flexible learning: participants can study while continuing to work as eye care professionals because they don't have to be online at the same time as their lecturers or fellow students. The main challenge with asynchronous learning is that students may feel disconnected from their teachers and from each other, but this can be addressed using the approaches described in this article (see panel).

**Figure:**
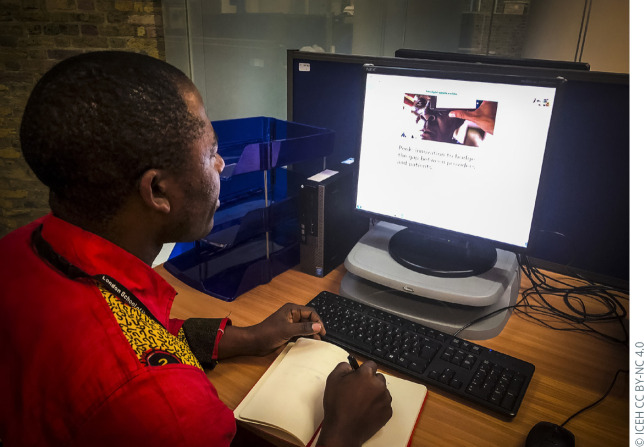
Programme manager and outreach coordinator Mathew Mbwogge uses ICEH's Global Blindness online course to update his knowledge. **CAMEROON**

## Learning platforms

The learning management systems or virtual learning environments used to organise and deliver content are also referred to as ‘learning platforms.’

Learning platforms are virtual ‘classrooms’ where you, as the educator, can post asynchronous material such as articles, videos, quizzes, and activities to structure student learning. Students can access these via a computer or their smartphone, even while travelling.

Learning platforms are most useful when there is a clear course structure that allows students to navigate through learning activities. All activities within the platform must be designed to be inclusive and accessible. This means that graphics, colours, and how content can be downloaded when there is poor access to the internet (so someone can work offline) are key considerations. Videos need to have transcripts and captioning (subtitles) so that people can read and follow. Text descriptions of visual material and transcripts of audio/video content are required so that people who have hearing and/or visual impairment can understand the content. Ideally, a platform should include discussion forums where students can share ideas and experiences and ask advice. This increases opportunities for interaction with educational content and fellow students, through ongoing discussions and reflection. A good learning platform should also enable educators to send emails to all students as one cohort.

The benefit of using an online learning platform is that educators can host all of the materials in one place, along with links to synchronous (live) learning activities such as lectures or workshop sessions, via Zoom or Microsoft Teams. The different tools that you can put onto a platform (such as quizzes, etc.) will vary, depending on the platform.

Educational institutions are beginning to invest in platforms such as Moodle, Blackboard, etc. but there are others which have been used such as Google. When selecting the platform and tools for interaction with students, it's always a good idea to speak with a learning technologist. Many universities now employ these specialists.

## Learner engagement

Interaction between educators and students (and among students) is very important, and often taken for granted in a classroom. In the online environment, interaction has to be planned and supported to create contact amongst students (peer-to-peer contact), and between students and educators (peer to educator contact).

**Examples of technologies for synchronous and asynchronous education**

**Synchronous technology**

**Online interaction tools**

**Poll Everywhere**, **Mentimeter** and **Padlet** are accessible via computer and smartphone and offer online polling, surveys, questions and answers, quizzes, and word clouds. They enable a variety of activity types that let educators visualise student responses in real time.

How to use them, and why: Use them for formative feedback during teaching to assess whether students understand key concepts and are attaining learning outcomes. Elements such as word clouds build visual consensus and encourage interaction and engagement. In Padlet, educators can create an online bulletin board where students can post their ideas and comment on each other's posts, in real time.

**Videoconferencing tools**

**Zoom** and **Microsoft Teams** are examples of online meeting or videoconferencing platforms. Both have automatic captioning, which transcribes and displays speech as people speak, and allow educators to create virtual ‘breakout rooms’ where students can have small group discussions.

How to use them, and why: Both Zoom and MS Teams allow remote attendance at lectures and tutorials and are fully inclusive for students with hearing impairment, thanks to the live captioning. Use breakout rooms to help students collaborate in smaller groups, which builds interpersonal relationship and helps to keep students engaged. Educators can ‘visit’ different groups during a breakout session, to see how students are doing. For more one-to-one support, one option is to open live sessions 30 minutes before a lecture is due to start so that students can arrive early and ask questions. Another option is to set up regular or weekly drop-in sessions. This will improve students’ sense of support from educators and help students who might be reluctant to speak up in larger groups.

**Other tools**

**Discord** and **Slack** offer topic-based channels for broadcasting live video or audiovisual materials and create an informal environment where students can collaborate with one another.

**OneDrive** and **Google Docs** enable educators and students to work on documents at the same time, either live or at their own pace, so they can be used for both synchronous and asynchronous learning.

**Asynchronous technology**

**Virtual Learning Environments**

**Moodle**, **BlackBoard** and **Google Classroom** are examples of virtual learning environments that allow educators to create a structured learning journey for their students. It provides access to resources such as reading materials, recordings (with transcripts), discussion forums, and interactive quizzes.

How to use them, and why: Use virtual learning environments to guide the students through the material in a structured way that allows them to build on existing knowledge and check their understanding as they progress from one section to the next. Students can interact with each other and with their educators on the discussion forum, which helps them to feel supported.

**Other tools**

**WhatsApp** groups allow students to create networks to support one another as they work through the content on the virtual learning environment. This helps to build a sense of community and aids peer-to-peer learning.

**Padlet**, **WordPress** and other blog websites can be used by students to publish group projects or to write individual reflections on their learning. Padlet, in particular, allows students to contribute their ideas asynchronously and comment on each other's contributions.

**Quizlet** is a useful online application that allows educators to create flash cards or study sets based on different topics supported by an artificial intelligence ‘learning assistant’. Educators can also encourage students to document their own learning by making their own sets of flash cards or study sets; this helps students with self-directed study.

It is essential for these interactions to be **timely** and **responsive**, so that the student knows the educator is present, and cares about them. Be mindful that every student learning online is working in a unique environment with its own stresses and challenges. By interacting with students, educators can bridge some gaps, motivate students, show understanding, and provide support – all of which reduces some of the inequities that students face. Educator facilitation as part of content delivery is a key factor in determining student engagement and performance.^1^ Alongside the content, it is always important that the digital skills of the students are developed. This is also known as ‘digital literacy’.

Online education is developing at a very fast pace and is likely to become a permanent part of the higher education experience. Therefore, the emphasis on developing online education is not driven by the technology; rather, online educators are harnessing technology to provide equitable and accessible opportunities for students and life-long learning.
